# Effect of Autologous Platelet-Rich Plasma Treatment on Refractory Thin Endometrium During the Frozen Embryo Transfer Cycle: A Pilot Study

**DOI:** 10.3389/fendo.2019.00061

**Published:** 2019-02-14

**Authors:** Hounyoung Kim, Ji Eun Shin, Hwa Seon Koo, Hwang Kwon, Dong Hee Choi, Ji Hyang Kim

**Affiliations:** Department of Obstetrics and Gynecology, Fertility Center of CHA Bundang Medical Center, CHA University School of Medicine, Seongnam, South Korea

**Keywords:** refractory thin endometrium, platelet-rich plasma, recurrent implantation failure, frozen embryo transfer, endometrial receptivity

## Abstract

**Objective:** Thin or damaged endometrium remains to be an unsolved problem in the treatment of patients with infertility. The empirical preference for endometrial thickness (EMT) among clinicians is >7 mm, and the refractory thin endometrium, which doesn't respond to standard medical therapies, can be the etiology of recurrent implantation failure (RIF). Autologous platelet-rich plasma (PRP) is known to help tissue regeneration and is widely used in various fields. In the present study, we conducted PRP treatment and investigated its effect on the refractory thin endometrium.

**Design:** Prospective interventional study (https://cris.nih.go.kr/cris, clinical trial registration number: KCT0003375).

**Methods:** Women who had a history of two or more failed IVF cycles and refractory thin endometrium were enrolled in this study. The main inclusion criteria were EMT of <7 mm after more than 2 cycles of previous medical therapy for increasing the EMT. Twenty-four women were enrolled in this study. The subjects were treated with intrauterine infusion of autologous PRP 2 or 3 times from menstrual cycle day 10 of their frozen-thawed embryo transfer (FET) cycle, and ET was performed 3 days after the final autologous PRP infusion. 22 patients underwent FET, and 2 patients were lost to follow up.

**Results:** The ongoing pregnancy rate and LBR were both 20%. The implantation and clinical pregnancy rates were 12.7 and 30%, respectively, and the difference was statistically significant. The average increase in the EMT was 0.6 mm compared with the EMT of their previous cycle. However, this difference was not statistically significant. Further, EMT of 12 patients increased (mean difference: 1.3 mm), while that of seven patients decreased (mean difference: 0.7 mm); the EMT of one patient did not change. There were no adverse effects reported by the patients who were treated with autologous PRP.

**Conclusions:** The use of autologous PRP improved the implantation, pregnancy, and live birth rates (LBR) of the patients with refractory thin endometrium. We assume that the ability of autologous PRP to restore the endometrial receptivity of damaged endometrium has some aspects other than increasing the EMT. The molecular basis of the treatment needs to be revealed in future studies.

## Introduction

Since the first introduction of *in vitro* fertilization and embryo transfer (IVF-ET), the technology has evolved rapidly, and the pregnancy rate with IVF-ET has significantly increased. However, thin or damaged endometrium remains to be an unsolved problem in the treatment of patients with infertility. Several treatments to restore endometrial receptivity have been attempted, including administration of exogenous estrogen, vitamin E, vaginal sildenafil citrate, and pentoxifylline (1–3). Patients with refractory thin endometrium who do not respond to the abovementioned treatment do not have many options, and an endometrium with a thickness below 7 mm is assumed as non-optimal for embryo implantation and is associated with a low pregnancy rate (4, 5). Recently, some progress in treating damaged or thin endometria has been made with the use of the cell proliferation method, including stem cell therapy (6, 7). However, there are still unsolved issues concerning the safety and usability of bone marrow-derived stem cells (8, 9).

Autologous platelet-rich plasma (PRP) is one alternative that is well-known for its safety. Such platelet products have been used since the 1970s, and they have become more popular since the 1990s (10). Platelets are known as the blood component that plays a crucial role in hemostasis. During the healing process, growth factors, cytokines, and chemokines are secreted from the α-granules inside platelets. The various secreted proteins have paracrine effects on myocytes (11), tendon cells (12), mesenchymal stem cells from different origins (13, 14), chondrocytes (15), osteoblasts (11, 16), fibroblasts (17), and endothelial cells (18), stimulating cell migration, cell proliferation, and angiogenesis and consequently inducing tissue regeneration (19). A study on a murine model was performed, which reported that intrauterine infusion of autologous PRP accelerated and enhanced regeneration of damaged endometria and that the fibrosis within decreased (20).

The first study on PRP for treating human thin endometrium *in vivo* was published in 2015 (21). Four studies followed and concluded that PRP is a potent treatment for thin endometri um (22–25). They stated that autologous PRP promotes endometrial growth and improves pregnancy outcomes. However, the number of patients was small, and they did not provide sufficient information on the type or concentration of PRP they used. It is known that the efficacy of PRP can vary according to the platelet concentration and cell component (19, 26). In the present study, we defined the platelet concentration and type of PRP that we used and investigated its effect on refractory thin endometrium regarding the pregnancy and live birth rates.

## Materials and Methods

### Study Population and Inclusion Criteria

We conducted an interventional prospective cohort study. Patients were recruited from December 2015 to June 2017 in a fertility center of a university hospital. Women who had a history of two or more failed IVF cycles and refractory thin endometrium were enrolled in this study. The inclusion criteria were as follows: (a) age of 20–45 years at the time of enrollment, (b) endometrial thickness (EMT) of <7 mm on the human chorionic gonadotropin (hCG) administration day in fresh ET cycles or on the end of estrogen priming day in frozen ET cycles in all of the previous cycles, (c) two or more failed IVF cycles, (d) more than two cycles of previous therapy for increasing the EMT, such as, hysteroscopic adhesiolysis following hormone replacement therapy, high dose estradiol valerate, transvaginal sildenafil administration, or pentoxifyilline combination with vitamin E, (f) frozen embryo available for ET, and (g) informed consent form signed. The exclusion criteria were as follows: (a) hematologic disorders, hemoglobin level of <9.0 g/dL or platelet count of <100,000/μL, (b) auto-immune disease, (c) chromosomal abnormality in the patient or spouse, (d) peripheral NK cell proportion of ≥12%, (e) body mass index (BMI) of ≥30 kg/m^2^, and (f) uncontrolled endocrine or other medical conditions, such as prolactinemia or thyroid diseases.

### Autologous PRP Preparation

On each PRP administration day, 18 mL of venous blood was drawn from the patients using 30 mL syringes coated with 2 cc of acid citrate A, anticoagulant solution (ACD-A; Arya Mabna Tashkhis, Iran). The blood samples were then moved into an aseptic PRP centrifuge kit (PROSYS PRP; Prodizen, Korea) and centrifuged at 1017 G for 3 min. The buffy coat and plasma just above the buffy coat were collected, and 0.7–1.0 mL of PRP was produced and infused into uterine cavity. Based on the data provided by the manufacturer, the platelet concentration of PRP ranged from 717 × 10^3^ to 1565 × 10^3^/μL, and the WBC concentration varied from 24,000 to 37,000/μL.

### Autologous PRP Administration and ET

Intrauterine autologous PRP administration was performed at the estrogen-primed FET cycle. The patients started to take a daily dose of 4–6 mg of estradiol valerate (Progynova; Bayer Schering Pharma, France) from menstrual cycle day (MCD) 2 to prepare the endometrium. The first autologous PRP infusion was performed on MCD 10 and was repeated at 3 day intervals until the EMT reached 7 mm. PRP was administered into the uterine cavity using an ET catheter within 1 h from completion of PRP preparation. The syringe containing the PRP was connected to ET catheter and the PRP was infused. Then the syringed filled with the air was used to push in the remaining PRP. Then the air bubble was confirmed in ultrasonography. Thereafter, the patients were prescribed with second-generation cephalosporin for 2 days as prophylaxis for infection. The maximum number of autologous infusions was limited to three.

Ultrasonography was performed to measure the EMT on MCD 2 and every autologous PRP administration day until ET. ET was conducted 3 days after the final autologous PRP administration. Luteal phase support was performed using either 90 mg of vaginal progesterone (Crinone gel 8%; Merck, Germany) or 50 mg of progesterone (Sugest Inj. 50 mg; Uni-Sankyo, India) administered via intramuscular injection daily from 3 days before the ET day. The serum β-hCG level was measured from peripheral blood 2 weeks after ET. Those with positive β-hCG results underwent ultrasonography another 2 weeks later to confirm clinical pregnancy. Clinical pregnancy was defined as the presence of intrauterine gestational sac. The luteal phase support was continued until 9 weeks of pregnancy. The obstetric progress of the pregnant patients was followed up via a timely chart review.

### Comparison of the Outcomes Between the Treatment and Previous Cycles

The variables of the most recent ET cycles were compared with those of the treatment cycle. The primary outcomes were the ongoing pregnancy rate and LBR. The secondary outcomes were the implantation rate, clinical pregnancy rate, and EMT increment compared with those on the previous cycle.

### Data Analysis

The statistical analysis was performed using the IBM SPSS® software, version 24 (IBM Corporation, Armonk, NY, USA). Wilcoxon signed-rank test was used to compare the differences between the pre-PRP and post-PRP EMT. A *P* value of < 0.05 was considered statistically significant. The implantation rate, clinical pregnancy rate, and live birth rate were analyzed using Fisher's exact test.

### Ethics Approval

This study was approved by the Institutional Review Board committee of Bundang CHA Medical Center.

## Results

### Study Population and Baseline Characteristics

A total of 24 women were recruited, and 22 of them underwent ET. One patient underwent preimplantation genetic screening, and all embryos were abnormal. Another patient had withdrawn owing to personal reasons. Among the 22 patients who underwent ET, two patients were lost to follow-up, and the data of the 20 remaining women were collected.

The average age of the patients was 38.4 years. The mean duration of infertility in the 20 women was 5.7 years, and the mean number of dilatation and evacuation performed was 1.3. The mean number of failed IVF cycle was 2.7. The mean EMT on the previous-cycle hCG administration or the final estrogen priming day was 5.4 mm. Sixteen of them were diagnosed with endometrial sclerosis or adhesion via hysteroscopy; the cause was radiation therapy for treating colon cancer in one patient and pelvic tuberculosis in another patient (Table 1).

**Table 1:** The baseline characteristics of the patients.

** 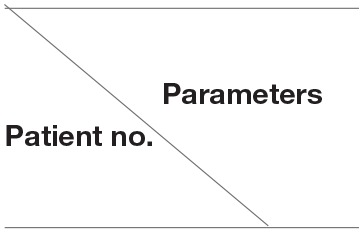 **	**Age**	**BMI (kg/m^**2**^)**	**Infertility factor**	**No. of D&E**	**Failed IVF cycles**	**Infertility duration (years)**	**Parity^#^**				**Hystero-scopic finding**	**Medical history**	**EMT (mm), On previous cycle hCG day or final priming day**
							**T**	**P**	**A**	**L**			
1	31	20.1	Tubal, IUA	0	2	3.1	0	0	0	0	IUA	Past Tuberculosis	5.8
2	35	28.4	Tubal, IUA	4	2	6	0	0	4	0	Synechia		4.8
3	39	20.7	IUA	2	2	7.1	0	0	2	0	Central IUA		6.7
4	40	23.6	Tubal DOR IUA	3	2	10.5	0	0	3	0	Erythematous EM		6.0
5	30	17.7	MF, IUA	1	2	1.9	0	0	1	0	No specific	Past PID	6.4
6	34	22.3	Tubal, IUA	0	3	6	0	0	0	0	Severe IUA		4.9
7	45	25.3	DOR,	1	3	8	0	0	1	0	No specific		5.2
8	33	22.4	IUA	1	2	3.7	0	0	1	0	Synechia		5.5
9	35	22.3	Tubal, IUA	0	4	6.5	0	0	1	0	Severe IUA		4.9
10	36	20.8	IUA	1	5	8.5	0	0	1	0	Synechia		5.5
11	37	21.3	POI, IUA	0	2	3.3	0	0	0	0	Severe IUA	Past RT (colon ca.)	4.0
12	38	25.4	unexplained	0	2	10	0	0	1	0	No specific		4.8
13	39	26.0	unexplained	1	4	4	0	0	1	0	Sclerotic EM		5.8
14	39	28.6	SM myoma, PGD	0	3	5	1	0	0	1	Synechia		6.8
15	41	24.0	IUA, MF	0	4	4.3	0	0	0	0	Severe IUA		4.3
16	41	20.6	IUA	3	2	1.5	0	0	3	0	Synechia		5.3
17	43	28.6	DOR IUA	5	2	6.6	0	0	5	0	Septum c fistula		5.7
18	43	19.2	IUA	1	2	5.7	1	1	2	1	Sclerotic fundus		4.5
19	44	22.4	MF, IUA	2	3	9	0	0	2	0	Sclerotic walls		6.5
20	44	25.7	DOR	0	2	2.4	2	0	0	2	Synechia		5.4
Mean ± SD or explanation	38.4 ± 4.3	23.3 ± 3.1		1.3 ± 1.5	2.7 ± 0.9	5.7 ± 2.6	17 primary 3 secondary				16 patients with endometrial pathology	–	5.4 ± 0.8

*EMT, endometrial thickness; hCG, human chorionic gonadotropin; RT, radiation therapy; IUA, intrauterine adhesion; DOR, diminished ovarian reserve; POI, primary ovarian insufficiency; MF, male factor; SM, submucosal; D&E, dilatation and evacuation; T, term birth; P, preterm birth; A, Abortion; L, living birth*.

# *The abortion count of parity includes chemical abortion*.

### Treatment Outcome

The number of embryos transferred in each patient was 2 or 3. The cleavage stage embryo grading was performed using the qualification scale by Veeck (27). The blastocysts were graded using the Gardner grading system (28). A good-grade embryo was defined as a grade I or II cleavage stage embryo with six or more cells and blastocyst score of 3BB or higher. The morula was considered as a good-grade embryo. Seventeen patients had at least one good-grade embryo; however, three patients had only poor-grade cleavage embryos.

**Figure 1:**
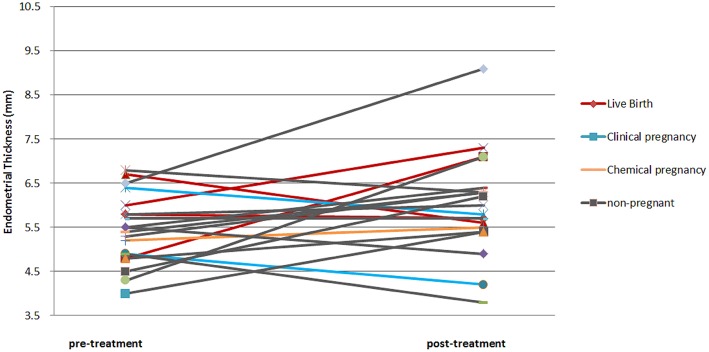
Pre- and post- endometrial thickness of each patient. The color of the line indicates the obstetric result of the patient.

The gestational sac was confirmed in 30% (*n* = 6) of the patients. One patient had missed abortion at 8+2 weeks of gestational age. Another patient had heterotopic pregnancy, and the intrauterine fetus was aborted at 6 weeks soon after laparoscopic removal of the ectopic conceptus. The live birth rate was 20% (*n* = 4). All the ongoing pregnancies resulted in live births without obstetric complications. The mean EMT after the PRP treatment was 6.0 mm. The average increment in the EMT was 0.6 mm. However, this difference was not statistically significant. Individually, the EMT of 12 patients increased (mean difference: 1.3 mm), while that of seven patients decreased (mean difference: 0.7 mm); however, the EMT of one patient did not change. Among the six clinical pregnancy cases, two were increased and four were decreased in EMT (Figure 1). There were no adverse effects reported by the patients. The outcomes of the treatment are summarized in Table 2.

**Table 2:** The results of autologous platelet-rich plasma treatment.

**Pat. No**.	**Obstetric result**		**EMT Final (mm)**		**Embryo grade and number**	**β-hCG^¥^**	**No. of G sac**
	**Week**	**Result**		**AVG**			
1	37+5	Live birth	5.7	6.4	10C GIII, 8C GI	+	1
2	38+6		7.1		Mor, 12C GII x 2.	+	1
3	38+3		5.6		Mor, 12C GI, 8C GI	+	2^*^
4	37+2		7.3		Mor, 12C GI, 10C GIII	+	1
5	8+2	Abortion	5.8	5.0	Mor, 10C GIII	+	1
6	6		4.2		Mor, 12C GII, 12C GIII	+	1^‡^
7	5	Chemical pregnancy	5.5	5.5	12C GII, 10C GI, 6C GIII	+	0
8	Not-pregnant	Not-pregnant	6.4	6.1	12C GI, 10C GI	–	N/A
9			3.8		Mor x 2, 12C GII	–	
10			4.9		12C GIV, 10C GIII, 10C GIV	–	
11			5.4		8C GII, 6C GIII, 4C GIII	–	
12			5.4		6C GIV x 2	–	
13			6.0		Mor, 12C GII, 10C GIII	–	
14			6.3		Mor x 2, 8C GIV	–	
15			7.1		8C GIII, 7C GIII, 6C GIII	–	
16			6.3		Mor x 2, 10C GIII	–	
17			5.7		12C GII, 12C GIII, 8C GI	–	
18			6.3		Mor, 8C GII, 4C GI	–	
19			9.1		Mor, 10C GII, 6C GIII	–	
20			6.2		Mor x 2, 10C GIII	–	
Average/	Full term: 4	LBR: 20%	6.0 ± 1.6			7 patients (35%)	6 patients
counts	patients (20%)	ABR: 15%				(35%)	(30%)

EMT, endometrial thickness; Mor, Morula; hCG, human chorionic gonadotropin; G sac, gestational sac; LBR, live birth rate; ABR, abortion rate;

¥ *β-hCG cut off: 35 mIU/mL*,

* *Vanishing twin*,

‡ *Missed abortion after laparoscopy for heterotopic pregnancy*.

### Comparison of the Outcomes Between the Treatment and the Previous Cycles

The treatment cycle outcomes were compared with the most recent ET cycle outcomes of each patient; the latter cycle was considered as the control cycle. The implantation, clinical pregnancy, and live birth rates in the treatment cycle were 12.7, 30, and 20%, respectively. The implantation, clinical pregnancy, and live birth rates in the control cycle were all 0%. The implantation and clinical pregnancy rates were significantly higher in the treatment cycle than in the control cycle. The age, BMI, number of transferred embryos, and number of good-grade embryos transferred were not significantly different. The comparison results are summarized in Table 3.

**Table 3:** Comparison of outcomes between the treatment and the previous cycles.

**Parameters / cycle**	**Previous Cycle**	**Treatment Cycle**	***P*-value**
Age	37.6 ± 4.4	38.4 ± 4.3	0.547
BMI (kg/m^2^)	22.89 ± 3.2	23.3 ± 3.1	0.640
EMT on hCG triggering or final preparation day^*^ (mm)	5.4 ± 0.8	6.0 ± 1.1	0.070
Cycle types (fresh/frozen)	10/10	0/20	
Number of transferred embryos	2.6 ± 0.7	2.8 ± 0.4	0.640
Number of good quality embryos transferred	1.7 ± 0.8	1.7 ± 0.9	0.967
Implantation rate (%)	0 (0/52)	12.7 (7/55)	0.015
Clinical pregnancy rate (%)	0 (0/20)	30 (6/20)	0.020
Ongoing pregnancy rate (%)	0 (0/20)	20 (4/20)	0.106
Live birth rate (%)	0 (0/20)	20 (4/20)	0.106

*EMT, endometrial thickness; hCG, human chorionic gonadotropin*.

* *EMT on hCG triggering day in fresh cycles and on final preparation day in frozen-thawed cycle*.

## Discussion

The purpose of the present study was to determine whether intrauterine administration of PRP would improve the pregnancy outcomes of patients with refractory thin endometrium. A total of 20 women were enrolled, and a clinical pregnancy rate of 30% and a live birth rate of 20% were achieved in these patients with poor prognosis. However, contrary to the expectation, even the mean EMT increased after treatment, and there was no association between the EMT changes and the ET outcomes.

Since the first study on *in vivo* autologous PRP on the human endometrium in 2015, five studies have been published (21–25). The inclusion criteria differed to some extent; however, all studies showed that autologous PRP is effective in repairing the damaged endometrium and improving the pregnancy outcomes. The LBRs reported by three studies were all above 25%. The autologous PRP preparation method and cell contents were not reported in three of the five studies. Table 4 summarizes the five previous studies on PRP for treating patients with repeated implantation failure owing to endometrial factors.

**Table 4:** Previous studies on autologous PRP treatment of human endometrium.

**References**	**Patients**			**PRP preparation**	**Result and Conclusion**				
	**No**	**HRT on FET**	**PRP repeat**		**EMT**	**β- hCG**	**On-going pregnancy**	**Live birth**	**Missed Ab**
Chang et al. (21)	5	Yes	2	L-PRP No information on platelet concentration	>7 mm (100%)	5 (100%)	4 (80%)	Not reported	1 (20%)
	EMT < 7 mm on previous hCG day despite HRT				PRP promote endometrial growth and improve pregnancy outcome				
Zadehmodarres et al. (25)	10	Yes	2	L-PRP No information on platelet concentration	>7 mm (100%)	5 (50%)	4 (40%)	Not reported	–
	EMT < 7 mm 4 patients were diagnosed as intrauterine adhesion by HSC				PRP is effective for endometrium growth				
Molina et al. (23)	19	Yes	2	No information on PRP preparation method, platelet concentration or WBC's in PRP	>9 mm (100%)	15 (73.7%)	5 (26.3%)	5 (26.3%)	1 (.26%)
	history of the refractory endometrium with at least 1 failed previous IVF cycle				PRP seems beneficial for endometrial microvasculature and endometrial receptivity of the refractory endometrium				
Colombo et al. (22)	8	–	–	No information	>6.5 mm (88%)	6 (85.7%)	4 (57%)	2 (28.5%)	1 (14.3%)
	more than 3 canceled FET d/t EMT < 6 mm HSC: no EM pathology				Inefficient expression of adhesion molecules can be replaced by PRP				
Tandulwadkar et al. (24)	68	Yes	2	No information on platelet concentration or WBC's in PRP	Average 7.22 mm	39 (60.9%)	31 (45.3%)	26 (38.2%)	5 (7.35%)
	suboptimal endometrial growth; thickness < 7 mm or < 5 vascular signals reaching central zone				Endometrial vascularity measured with power Doppler was increased				

*EMT, endometrial thickness; hCG, human chorionic gonadotropin; MCD, menstrual cycle day; HRT, hormonal replacement therapy; FET, frozen-thawed Embryo Transfer; HSC, hysteroscopy; L-PRP, Leucocyte-platelet rich plasma*.

Although PRP is widely applied in different clinical areas, the procedure in preparing PRP is not yet standardized. Therefore, the platelet quantification and growth factor contents are not defined (19). The previous studies did not present critical information on the PRP used, such as cell contents, platelet concentration, and activation. We attempted to provide information on PRP and its preparation method and searched for the best-known evidence to improve the effectiveness of PRP. The optimal biological effect seems to occur when PRP with a platelet concentration of approximately 1,000,000/μL (503,000–1,729,000/μL) is used. At lower concentrations, the effect is suboptimal, while higher concentrations might have a paradoxically inhibitory effect (29). We employed a PRP preparation method using an aseptic PRP preparation kit that had manufacturer's information on the platelet count of the final product as 717,000 to 1,565,000/μL and the WBC concentration as 24,000 to 37,000/μL.

There are four categories of platelet concentrate preparations: leukocyte-poor or pure PRP (P-PRP), leukocyte PRP (L-PRP), pure platelet-rich fibrin clot, and leukocyte platelet-rich fibrin clot. Among them, two families contain a significant number of leukocytes. P-PRP and pure platelet-rich fibrin clot are made without the buffy coat and considered to contain a minimal amount of leukocytes (30). The variety of PRP preparations currently available on the market has led to considerable confusion in the evaluation of the potential clinical benefits of PRP in different applications (26). The advantage of each type of PRP in specific tissues has not been defined yet.

Two of the previous studies (21, 25) provided information that they used the buffy coat of the centrifuge, and this implies that they employed L-PRP. There are conflicting opinions on the leukocyte content in PRP. One view is that leukocytes increase inflammation and reduce tissue regeneration (31). Another view is that inflammation is an essential step in the healing process (32), especially for protection against infection and clearance of tissue debris (33). There was also a recent study by Cousins et al. that provided evidence that mononuclear phagocytes have roles in scar-less endometrial healing in menstrual cycles (34). We also used the buffy coat of the centrifuge, and thus, L-PRP was employed. The leukocytes in PRP could have increased inflammation; however, the implantation and pregnancy rates improved. Since no studies have stated the use of P-PRP, its effectiveness needs to be explored in the future.

LBR was reported in two of the previous studies (23, 24). The first study reported 26.3% of live birth after PRP treatment and the LBR of the second study was 38.2%. The difference of LBR between the previous and the present studies may be caused by the difference in patient characteristics. The inclusion criteria of the first study was “aged between 33 and 45 years with a previous history of refractory endometrium and at least one failed IVF attempt” and the second study criteria was “between 22 and 40 years of age with a suboptimal endometrial pattern, as identified by ET <7 mm despite standard dose of estradiol valerate, or suboptimal endometrial vascularity, defined as <5 vascular signals reaching the central zone (zones 3 and 4 as per Applebaum grading) of the endometrium.” The patients of our study had at least 2 failed previous IVF cycles and had no improvement in endometrial thickness after two or more cycles of medical therapies. The average of infertile period was 5.7 years and more than 2/3 of them had intrauterine adhesion from hysteroscopic findings.

The EMT was reported to have increased after PRP treatment in the previous studies. In the present study, the average increase in the EMT was 0.6 mm. However, this difference was not statistically significant. Furthermore, there was no correlation between the EMT increase and pregnancy outcomes. Among the six clinical pregnancy cases, two were increased, and four were decreased in EMT. A study examining the pregnancy outcomes of euploid ET (35) and a systemic review with meta-analysis on the EMT as a prognostic factor of pregnancy (36) reported that the EMT was not significantly associated with the pregnancy outcomes. Accordingly, we assumed that autologous PRP intrauterine administration improved the endometrial receptivity of the patients with refractory endometrium through the way that cannot be checked by EMT.

There was no difference in other clinical characteristics including age, infertility duration, number of failed IVF cycles, and transferred embryo number and grade according to pregnancy outcomes. Therefore, there is no prognostic factors expecting successful results in PRP treatment. However, this result might be due to small number of cases and further study with larger number of subjects is necessary to confirm this finding.

Endometrial receptivity is controlled by dynamic and precise molecular and cellular events of cytokines, homeobox transcription factors, and genes (37). Of the cytokines, leukemia inhibitory factor (LIF) has been found to have a role in uterine preparation and embryo attachment (38, 39). *Lif*-deficient female mice showed an implantation failure and were rescued with LIF supplementation (40, 41). PRP treatment upregulates LIF expression in endometrial stromal cells (42), and upregulated LIF expression could enhance endometrial receptivity. It is also suggested that PRP may exert some effect to enhance the placentation of trophoblasts. Amable et al. showed that the levels of 12 proteins increased in activated PRP in comparison with whole blood plasma or platelet-poor plasma. Six growth factors (i.e., PDGF-AA, PDGF-AB, PDGF-BB, TGF-β1, TGF-β2, and EGF), three anti-inflammatory cytokines (i.e., IL-4, IL-13, and IFN-α), and three pro-inflammatory cytokines (i.e., IL-8, IL-17, and TNF-α) were included (19). These cytokines and growth factors may increase endometrial receptivity. The vascularity of the endometrium increased in the study by Tandulwadkar et al. The endometrial vascularity measured using power doppler after PRP treatment significantly increased, especially in the group that achieved pregnancy after PRP treatment (24). More studies on the molecular basis of PRP treatment are required to reveal the exact mechanism and to specify which group of patients would benefit the most from the autologous PRP treatment of the endometrium.

Prevention of intrauterine adhesion after curettage or hysteroscopic operation of myoma or endometrial polyp is a good candidate for endometrial PRP treatment. However, concerns have been raised regarding PRP use for regeneration or reconstruction on cancer tissue removal site because PRP contains and induces various growth factors and cytokines to promote cell proliferation and regeneration. There have been a few clinical studies reporting favorable outcomes of using PRP in breast reconstruction after mastectomy in breast cancer patients (43, 44). However, there is no study on endometrial PRP treatment after curettage in endometrial cancer patients. Although *in vitro* studies reported that the growth factors and VEGF of PRP could promote cancer recurrence (45, 46), the role of PRP in tumor proliferation and recurrence in cancer patients yet needs further investigation.

In the present study, the PRP treatment was performed during the FET cycle; however, half of the most recent cycles that were used as control cycles were conducted during the fresh cycle. It is still controversial whether FET increases the pregnancy rate in IVF-ET. In a recent large-scale prospective randomized clinical trial (RCT), Shi et al. reported that there is no significant difference in the pregnancy outcomes between fresh and frozen embryos when transferred to ovulatory women (47). Further, a meta-analysis including four RCTs also showed that there is no clear evidence on the difference in the cumulative pregnancy rates between fresh and frozen-thawed ET cycles (48). In the present study, 14 of the 20 patients have undergone FET in the previous cycles, and all the cycles failed to achieve pregnancy. Among the six pregnant cases after the PRP treatment, three underwent fresh ET, and the other three underwent FET as the control cycle. Therefore, we assumed that the difference in the transfer cycle characteristics (fresh vs. frozen) would not affect the outcomes significantly in our study.

There are limitations in this study. First, the study population was small to show a statistically significant result on live birth rate. The live birth rate was 20% in the treatment cycles, but was not significantly increased compared with that in the control cycles showing no pregnancy. A follow-up study consisting of larger number of patients is necessary and is actually currently being performed. Second, this study was not an RCT; thus, the effectiveness of the PRP treatment was shown only by comparison with the most recent previous cycle of each patient.

The present study was conducted as a pilot study to determine the effects of autologous PRP treatment on refractory thin endometrium. The implantation, clinical pregnancy and live birth rates reached up to 12.7, 30, and 20%, respectively. This result is a noticeable improvement considering the patients' history. Further studies on the molecular basis of this PRP treatment and well-designed RCTs are necessary to reveal the exact mechanism and to obtain more solid evidence on the beneficial effect of PRP on the endometrium of various pathophysiology.

## Author Contributions

HKi: collection, analysis, and interpretation of data, drafting, and revision of the manuscript; JS, HKo, HKw, and DC: conception and design, data interpretation, and revision of the manuscript; JK: conception and design, data analysis, data interpretation, revision and final approval of the manuscript.

### Conflict of Interest Statement

The authors declare that the research was conducted in the absence of any commercial or financial relationships that could be construed as a potential conflict of interest.
